# Risk mapping of clonorchiasis in the People’s Republic of China: A systematic review and Bayesian geostatistical analysis

**DOI:** 10.1371/journal.pntd.0005239

**Published:** 2017-03-02

**Authors:** Ying-Si Lai, Xiao-Nong Zhou, Zhi-Heng Pan, Jürg Utzinger, Penelope Vounatsou

**Affiliations:** 1 Swiss Tropical and Public Health Institute, Basel, Switzerland; 2 University of Basel, Basel, Switzerland; 3 National Institute of Parasitic Diseases, Chinese Center for Disease Control and Prevention, Shanghai, People’s Republic of China; 4 WHO Collaborating Centre for Tropical Diseases, Key Laboratory of Parasite and Vector Biology, Ministry of Health, Shanghai, People’s Republic of China; 5 Tianjin Modern Vocational Technology College, Tianjin, People’s Republic of China; Australian National University, AUSTRALIA

## Abstract

**Background:**

Clonorchiasis, one of the most important food-borne trematodiases, affects more than 12 million people in the People’s Republic of China (P.R. China). Spatially explicit risk estimates of *Clonorchis sinensis* infection are needed in order to target control interventions.

**Methodology:**

Georeferenced survey data pertaining to infection prevalence of *C*. *sinensis* in P.R. China from 2000 onwards were obtained via a systematic review in PubMed, ISI Web of Science, Chinese National Knowledge Internet, and Wanfang Data from January 1, 2000 until January 10, 2016, with no restriction of language or study design. Additional disease data were provided by the National Institute of Parasitic Diseases, Chinese Center for Diseases Control and Prevention in Shanghai. Environmental and socioeconomic proxies were extracted from remote-sensing and other data sources. Bayesian variable selection was carried out to identify the most important predictors of *C*. *sinensis* risk. Geostatistical models were applied to quantify the association between infection risk and the predictors of the disease, and to predict the risk of infection across P.R. China at high spatial resolution (over a grid with grid cell size of 5×5 km).

**Principal findings:**

We obtained clonorchiasis survey data at 633 unique locations in P.R. China. We observed that the risk of *C*. *sinensis* infection increased over time, particularly from 2005 onwards. We estimate that around 14.8 million (95% Bayesian credible interval 13.8–15.8 million) people in P.R. China were infected with *C*. *sinensis* in 2010. Highly endemic areas (≥ 20%) were concentrated in southern and northeastern parts of the country. The provinces with the highest risk of infection and the largest number of infected people were Guangdong, Guangxi, and Heilongjiang.

**Conclusions/Significance:**

Our results provide spatially relevant information for guiding clonorchiasis control interventions in P.R. China. The trend toward higher risk of *C*. *sinensis* infection in the recent past urges the Chinese government to pay more attention to the public health importance of clonorchiasis and to target interventions to high-risk areas.

## Author summary

Clonorchiasis is an important food-borne trematodiases and it has been estimated that more than 12 million people in China are affected. Precise information on where the disease occurs can help to identify priority areas for where control interventions should be implemented. We collected data from recent surveys on clonorchiasis and applied Bayesian geostatistical models to produce model-based, high-resolution risk maps for clonorchiasis in China. We found an increasing trend of infection risk from 2005 onwards. We estimated that approximately 14.8 million people in China were infected with *Clonorchis sinensis* in 2010. Areas where the high prevalence of *C*. *sinensis* was predicted were concentrated in the provinces of Guangdong, Guangxi, and Heilongjiang. Our results suggest that the Chinese government should pay more attention on the public health importance of clonorchiasis and that specific control efforts should be implemented in high-risk areas.

## Introduction

Clonorchiasis is an important food-borne trematodiases in Asia, caused by chronic infection with *Clonorchis sinensis* [1,2]. Symptoms of clonorchiasis are related to worm burden; ranging from no or mild non-specific symptoms to liver and biliary disorders [3,4]. *C*. *sinensis* is classified as a carcinogen [5], as infection increases the risk of cholangiocarcinoma [6]. Conservative estimates suggest that around 15 million people were infected with *C*. *sinensis* in 2004, over 85% of whom were concentrated in the People’s Republic of China (P.R. China) [6–8]. It has also been estimated that, in 2005, clonorchiasis caused a disease burden of 275,000 disability-adjusted life years (DALYs), though light and moderate infections were excluded from the calculation [9].

Therefore, two national surveys have been conducted for clonorchiasis in P.R. China; the first national survey was done in 1988–1992 and the second national survey in 2001–2004. Of note, the two surveys used an insensitive diagnostic approach with only one stool sample subjected to a single Kato-Katz thick smear. The first survey covered 30 provinces/autonomous regions/municipalities (P/A/M) with around 1.5 million people screened, and found an overall prevalence of 0.37% [10]. Data from the second survey, which took place in 31 P/A/M and screened around 350,000 people, showed an overall prevalence of 0.58% [7]. Another dataset in the second national survey is a survey pertaining to clonorchiasis conducted in 27 endemic P/A/M using triplicate Kato-Katz thick smears from single stool samples. The overall prevalence was 2.4%, corresponding to 12.5 million infected people [8]. Two main endemic settings were identified; the provinces of Guangdong and Guangxi in the south and the provinces of Heilongjiang and Jilin in the north-east [1,2,6]. In the latter setting, the prevalence was especially high in Korean (minority) communities. In general, males showed higher infection prevalence than females and the prevalence increased with age [6,8].

The life cycle of *C*. *sinensis* involves specific snails as first intermediate hosts, freshwater fish or shrimp as the second intermediate host, and humans or other piscivorous mammals as definitive hosts, who become infected through consumption of raw or insufficiently cooked infected fish [1,2,11,12]. Behavioral, environmental, and socioeconomic factors that influence the transmission of *C*. *sinensis* or the distribution of the intermediate hosts affect the endemicity of clonorchiasis. For example, temperature, rainfall, land cover/usage, and climate change that affect the activities and survival of intermediate hosts, are considered as potential risk factors [13,14]. Socioeconomic factors and consumption of raw freshwater fish are particularly important in understanding the epidemiology of clonorchiasis [15]. Consumption of raw fish dishes is a deeply rooted cultural practice in some areas of P.R. China, while in other areas it has become popular in recent years, partially explained by perceptions that these dishes are delicious or highly nutritious [1,2,16,17].

Treatment with praziquantel is one of the most important measures for the management of clonorchiasis, provided to infected individuals or entire at-risk groups through preventive chemotherapy [18,19]. Furthermore, information, education, and communication (IEC), combined with preventive chemotherapy, is suggested for maintaining control sustainability [20]. Elimination of raw or insufficiently cooked fish or shrimp is an effective way for prevention of infection, but this strategy is difficult to implement due to deeply rooted traditions and perceptions [1]. Environmental modification is an additional way of controlling clonorchiasis, such as by removing unimproved lavatories built adjacent to fish ponds in endemic areas, thus preventing water contamination by feces [1,21].

Maps displaying where a specific disease occurs are useful to guide prevention and control interventions. To our knowledge, only a province-level prevalence map of *C*. *sinensis* infection is available for P.R. China, while high-resolution, model-based risk estimates based on up-to-date survey data are currently lacking [1]. Bayesian geostatistical modeling is a rigorous inferential approach to produce risk maps. The utility of this method has been demonstrated for a host of neglected tropical diseases, such as leishmaniasis, lymphatic filariasis, schistosomiasis, soil-transmitted helminthiasis, and trachoma [22–28]. The approach relies on the qualification of the association between disease risk at observed locations and potential risk factors (e.g., environmental and socioeconomic factors), thus predicting infection risk in areas without observed data [28]. Random effects are usually introduced to the regression equation to capture the spatial correlation between locations via a spatially structured Gaussian process [26].

Here, we compiled available survey data on clonorchiasis in P.R. China, identified important climatic, environmental, and socioeconomic determinants, and developed Bayesian geostatistical models to estimate the risk of *C*. *sinensis* infection at high spatial resolution throughout the country.

## Methods

### Ethics statement

This work is based on clonorchiasis survey data extracted from the peer-reviewed literature and national surveys in P.R. China. All data were aggregated and do not contain any information at individual or household levels. Hence, there are no specific ethical issues that warranted attention.

### Disease data

A systematic review was undertaken in PubMed, ISI Web of Science, China National Knowledge Internet (CNKI), and Wanfang Data from January 1, 2000 until January 10, 2016 to identify studies reporting community, village, town, and county-level prevalence data of clonorchiasis in P.R. China. The search terms were “clonorchi*” (OR “liver fluke*”) AND “China” for Pubmed and ISI Web of Science, and “huazhigaoxichong” (OR “ganxichong”) for CNKI and Wanfang. Government reports and other grey literature (e.g., MSc and PhD theses, working reports from research groups) were also considered. There were no restrictions on language or study design. County-level data on clonorchiasis collected in 27 endemic P/A/M in the second national survey were provided by the National Institute of Parasitic Diseases, Chinese Center for Disease Control and Prevention (NIPD, China CDC; Shanghai, P.R. China).

Titles and abstracts of articles were screened to identify potentially relevant publications. Full text articles were obtained from seemingly relevant pieces that were screened for *C*. *sinensis* infection prevalence data. Data were excluded if they stemmed from school-based surveys, hospital-based surveys, case-control studies, clinical trials, drug efficacy studies, or intervention studies (except for baseline or control group data). Studies on clearly defined populations (e.g., travellers, military personnel, expatriates, nomads, or displaced or migrating populations) that were not representative of the general population were also excluded. We further excluded data based on direct smear or serum diagnostics due to the known low sensitivity or the inability to differentiate between past and active infection, respectively. All included data were georeferenced and entered into the open-access Global Neglected Tropical Diseases (GNTDs) database [29].

### Environmental, socioeconomic, and demographic data

Environmental, socioeconomic, and demographic data were obtained from different accessible data sources (Table 1). The data were extracted at the survey locations and at the centroids of a prediction grid with grid cells of 5×5 km spatial resolution. Land cover data were re-grouped to the following five categories: (i) forests, (ii) scrublands and grass, (iii) croplands, (iv) urban, and (v) wet areas. They were summarized at each location (of the survey or grid cell) by the most frequent category over the period 2001–2004 for each pixel of the prediction grid. Land surface temperature (LST) and normalized difference vegetation index (NDVI) were averaged annually. We used human influence index (HII), urban extents, and gross domestic product (GDP) per capita as socioeconomic proxies. The latter was obtained from the P.R. China yearbook full-text database at county-level for the year 2008 and georeferenced for the purpose of our study. Details about data processing are provided in Lai *et al*. [26]. We georeferenced surveys reporting aggregated data at county level by the county centroid and linked them to the average values of our covariates within the specific county. The mean size of the corresponding counties was around 2,000 km^2^.

**Table 1:** Remote sensing data sources^a^.

Source	Data type	Data period	Temporal resolution	Spatial resolution
MODIS/Terra^b^	LST^j^	2001–2015	8 days	1 km
MODIS/Terra^b^	NDVI^k^	2001–2015	16 days	1 km
MODIS/Terra^b^	Land cover	2001–2004	Yearly	1 km
WorldClim^c^	Elevation	2000	-	1 km
WorldClim^c^	Precipitation	1950–2000	Monthly	1 km
SWBD^d^	Water bodies	2000	-	30 m
ISRIC^f^	Soil pH	-	-	10 km
Atlas of the Biosphere^g^	Soil-moisture	1950–1999	-	50 km
SEDAC^h^	Population data	2010	-	5 km
SEDAC^h^	HII^l^	1995–2004	-	1 km
SEDAC^h^	Urban extents	1990–2000	-	1 km
China Yearbook^i^	GDP per capita	2008	-	County-level

^a^Land cover data accessed on June 1, 2011 and other data accessed on January 1, 2016.

^b^Moderate Resolution Imaging Spectroradiometer (MODIS)/Terra, available at: https://lpdaac.usgs.gov/.

^c^Available at: http://www.worldclim.org/current.

^d^Shuttle Radar Topography Mission Water Body Data (SWBD), available at: http://gis.ess.washington.edu/data/vector/worldshore/index.html.

^f^International Soil Reference and Information Center, available at: http://www.isric.org/data/isric-wise-derived-soil-properties-5-5-arc-minutes-global-grid-version-12.

^g^Available at: http://nelson.wisc.edu/sage/data-and-models/atlas/data.php?incdataset=Soil%20Moisture.

^h^Socioeconomic Data and Applications Center, available at: http://sedac.ciesin.org/.

^i^China yearbook full-text database, available at: http://acad.cnki.net/Kns55/brief/result.aspx?dbPrefix=CYFD.

^j^Land surface temperature, day and night.

^k^Normalized difference vegetation index.

^l^Human influence index.

### Statistical analysis

We grouped survey years into two categories (before 2005 and from 2005 onwards). We selected 2005 as the cutoff year because after the second national survey on important parasitic diseases in 2001–2004, the Chinese government set specific disease control targets and launched a series of control strategies [7,30]. We standardized continuous variables to mean zero and standard deviation one (SD = 1). We calculated Pearson’s correlation between continuous variables and dropped one variable among pairs with correlation coefficient greater than 0.8 to avoid collinearity, which can lead to wrong parameter estimation [31]. Researchers have suggested different correlation thresholds of collinearity ranging from 0.4 to 0.85 [31]. To test the sensitivity of our threshold, we also considered two other thresholds, i.e., 0.5 and 0.7. Three sets of variables were obtained corresponding to the three thresholds and were used separately in the variable selection procedure. Furthermore, continuous variables were converted to two- or three-level categorical ones according to preliminary, exploratory, graphical analysis.

We carried out Bayesian variable selection to identify the most important predictors of the disease risk. In particular, we assumed that the number of positive individuals *Y*_*i*_ arises from a binominal distribution *Y*_*i*_∼*Bn*(*p*_*i*_, *n*_*i*_), where *n*_*i*_ and *p*_*i*_ are the number of individuals examined and the probability of infection at location *i* (*i* = 1,2,…,*L*), respectively. We modeled the covariates on the logit scale, that is $logit(p_{i})=\beta_{0}+\sum_{k=1}\beta_{k}\times X_{i}^{\left(k\right)}$, where ***β***_***k***_ is the regression coefficient of the *k*^*th*^ covariate ***X***^(***k***)^. For a covariate in categorical form, ***β***_***k***_ is a vector of coefficients {*β*_*kl*_}, *l* = 1,…,*M*_*k*_, where *M*_*k*_ is the number of categories, otherwise it has a single element *β*_*k*0_. We followed a stochastic search variable selection approach [32], and for each predictor ***X***^(***k***)^ we introduced a categorical indicator parameter *I*_*k*_ which takes values *j*, *j* = 0,1,2 with probabilities *π*_*j*_ such that *π*_0_ + *π*_1_ + *π*_2_ = 1. *I*_*k*_ = 0 indicates exclusion of the predictor from the model, *I*_*k*_ = 1 indicates inclusion of ***X***^(***k***)^ in linear form and *I*_*k*_ = 2 suggests inclusion in categorical form. We adopted a mixture of Normal prior distribution for the parameters *β*_*k*0_, known as spike and slab prior, proposing a non-informative prior $\beta_{k0}∼N(0,\sigma_{B}^{2})$ with probability *π*_1_ in case ***X***^(***k***)^ is included in the model (i.e., *I*_*k*_ = 1) in linear form (slab) and an informative prior $\beta_{k0}∼N(0,\vartheta_{0}\sigma_{B}^{2})$ with probability (1 − *π*_1_), shrinking *β*_*k*0_ to zero (spike) if the linear form is excluded from the model. *ϑ*_0_ is a constant, fixed to a small value i.e., *ϑ*_0_ = 0.00025 forcing the variance to be close to zero. In a formal way the above prior is written $\beta_{k0}∼\delta_{1}(I_{k})N(0,\sigma_{B}^{2})+(1-\delta_{1}(I_{k}))N(0,{\vartheta_{0}\sigma }_{B}^{2})$ where *δ*_*j*_(*I*_*k*_) is the Dirac function taking the value 1 if *I*_*k*_ = *j* and zero otherwise. Similarly, for the coefficients {*β*_*kl*_}, *l* = 1,…,*M*_*k*_ corresponding to the categorical form of ***X***^(***k***)^ with *M*_*k*_ categories, we assume that $\beta_{kl}∼\delta_{2}(I_{k})N(0,\sigma_{Bl}^{2})+(1-\delta_{2}(I_{k}))N(0,{\vartheta_{0}\sigma }_{Bl}^{2})$. For the inclusion/exclusion probabilities *π*_*j*_, we adopt a non-informative Dirichlet prior distribution, i.e. (*π*_0_, *π*_1_, *π*_2_)^*T*^∼*Dirichlet*(3,***a***), ***a*** = (1,1,1)^*T*^. We also used non-informative inverse gamma prior distributions, *IG*(2.01,1.01) for the variance hyperparameters $\sigma_{B}^{2}$ and $\sigma_{Bl}^{2},\;l=1,\ldots ,M_{k}$. We considered as important, those predictors with posterior inclusion probabilities of *π*_*j*_ greater than 50%. The above procedure fits all models generated by all combinations of our potential predictors and selects as important those predictors which are included in more than 50% of the models.

Bayesian geostatistical logistic regression models were fitted on *C*. *sinensis* survey data to obtain spatially explicit estimates of the infection risk. The predictors selected from the variable selection procedure were included in the model. The model extended the previous formulation by including location random effects on the logit scale, that is $logit(p_{i})=\beta_{0}+\sum_{k=1}\beta_{k}\times X_{i}^{\left(k\right)}+\varepsilon_{i}$, where covariate ***X***^(***k***)^ are the predictors (with functional forms) that have been identified as important in the variable selection procedure. We assumed that location-specific random effects ***ε*** = (*ε*_1_,…,*ε*_*L*_)^*T*^ followed a multivariate normal prior distribution ***ε***∼*MVN* (0, Σ), with exponential correlation function $\Sigma_{ij}=\sigma_{sp}^{2}exp(-\rho d_{ij})$, where *d*_*ij*_ is the Euclidean distance between locations, and *ρ* is the parameter corresponding to the correlation decay. We also considered non-informative normal prior distributions for the regression coefficient $\beta_{kl,l=0,1,\ldots ,M_{k}}$, that is *β*_*kl*_∼*N*(0, 10^2^), an inverse gamma prior distribution for the spatial variance $\sigma_{sp}^{2}∼IG(2.01,1.01)$, and a gamma prior for the correlation decay *ρ*∼*G*(0.01,0.01). We estimated the spatial range as the minimum distance with spatial correlation less than 0.1 equal to −log(0.1)/*ρ*. We formulated the model in a Bayesian framework and applied Markov chain Monte Carlo (MCMC) simulation to estimate the model parameters in Winbugs version 1.4 (Imperial College London and Medical Research Council; London, United Kingdom) [33]. We assessed convergence of sampling chains using the Brooks-Gelman-Rubin diagnostic [34].

We fitted the model on a random subset of 80% of survey locations and used the remaining 20% for model validation. Mean error and the percentage of observations covered by 95% Bayesian credible intervals (BCIs) of posterior predicted prevalence were calculated to access the model performance. Bayesian kriging was employed to predict the *C*. *sinensis* infection risk at the centroids of a prediction grid over P.R. China with grid cells of 5 × 5 km spatial resolution [35]. This spatial resolution is often used for estimation of disease risk across large regions as it is a good trade-off between disease control needs and computational burden. Furthermore, predictions become unreliable when the grid cells have higher resolution than that of the predictors used in the model. Population-adjusted prevalence (median and 95% BCI) for each province was calculated using samples of size 500 from the predictive posterior distribution estimated over the gridded surface. These samples available for each grid cell were converted to samples from the predictive distribution of the population-adjusted prevalence for each province by multiplying them with the gridded population data, summing them over the grid cells within each province and divided them by the province population. The samples from the population-adjusted prevalence for each province were summarized by their median and 95% BCI.

Our disease data consist of point-referenced (village- or town-level) and areal (county-level) data. Analyses ignoring the areal data may loss valuable information, especially in regions where point-referenced data is sparse. Here, we assumed a uniform distribution of infection risk within each survey county and treated the areal data as point-referenced data by setting the survey locations as the centroids of the corresponding counties. To assess the effect of this assumption on our estimates, we simulated data over a number of hypothetical survey locations within the counties and compared predictions based on approaches using the county aggregated data together with the data at individual georeferenced survey locations and using the data at individual georeferenced survey locations only (excluded the county aggregated data). The former approach gave substantially better disease risk prediction compared to the later one. The methodology for the simulation study and its results are presented in Supplementary Information S1 Text and S1 Fig, respectively.

## Results

### Data summaries

**Fig 1:**
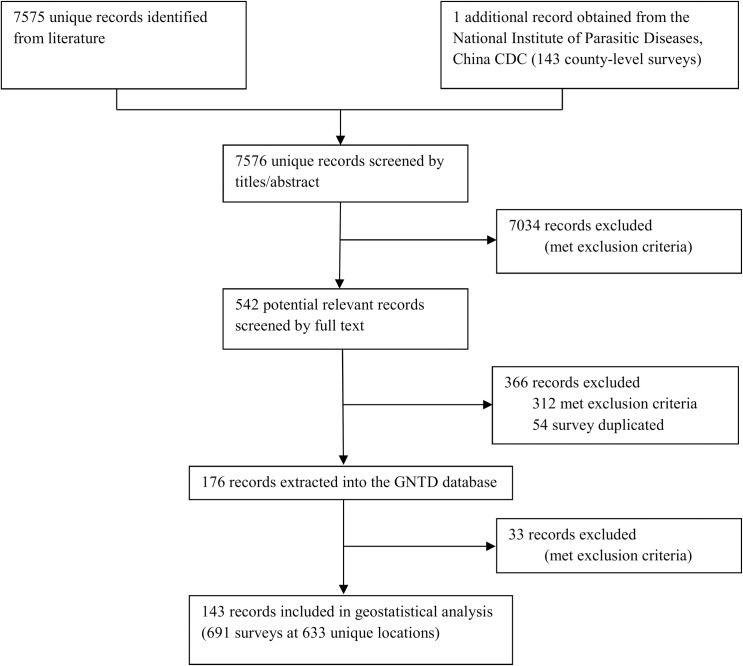
Data selection flow chart.

**Fig 2:**
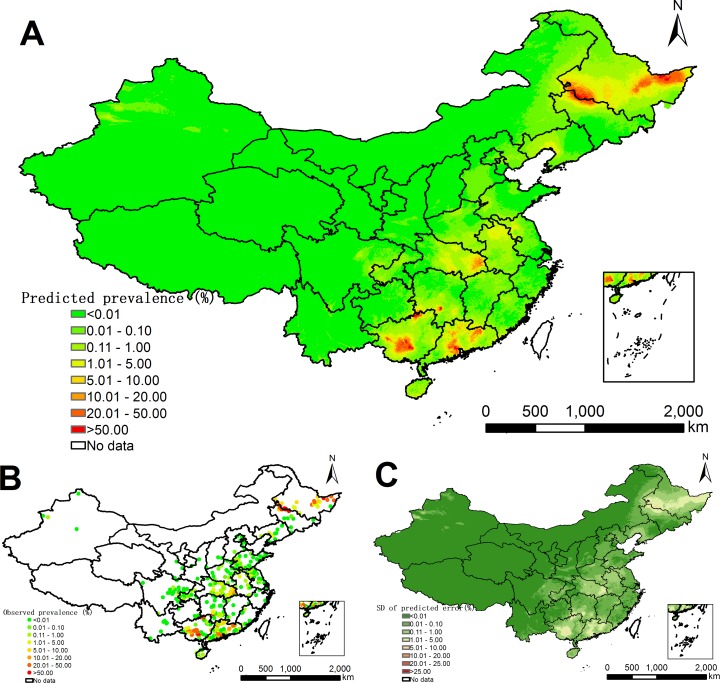
Model-based prediction risk maps of *C*. *sinensis* infection in P.R. China from 2005 onwards. (A) Predictive prevalence based on the median of the posterior predictive distribution of infection risk. (B) Survey locations and observed prevalence over P.R. China. (C) Prediction uncertainty based on the standard deviation of the posterior predictive distribution of infection risk.

A data selection flow chart for the systematic review is presented in Fig 1. We identified 7,575 records through the literature search and obtained one additional report provided by NIPD, China CDC (Shanghai, P.R. China). According to our inclusion and exclusion criteria, we obtained 143 records for the final analysis, resulting in 691 surveys for *C*. *sinensis* at 633 unique locations published from 2000 onwards. A summary of our survey data, stratified by province, is provided in Table 2. The geographic distribution of locations and observed *C*. *sinensis* prevalence are shown in Fig 2B. We obtained data from all provinces except Inner Mongolia, Ningxia, Qinghai, and Tibet. We collected more than 50 surveys in Guangdong, Guangxi, Hunan, and Jiangsu provinces. Over 45% of surveys were conducted from 2005 onwards. Around 90% of surveys used the Kato-Katz technique for diagnosis, while 0.14% of surveys had no information on the diagnostic technique employed. The overall raw prevalence, calculated as the total number of people infected divided by the total number of people examined from all observed surveys, was 9.7%.

**Table 2:** Overview of clonorchiasis survey data in P.R. China.

Province	Relevant papers	Total^a^	Raw prevalence (%)	Location type^a^		Period	Year of survey^a^		Diagnostic method (%)^b^					
				Point	County		2000-2004	≥2005	KK	Flot	Digest	Conc	Sedi	NS
Anhui	3	12/12	0.65	1/1	11/11	2001-2014	11/11	1/1	100.0	0.0	0.0	0.0	0.0	0.0
Beijing	1	3/3	0.00	0/0	3/3	2004-2004	3/3	0/0	100.0	0.0	0.0	0.0	0.0	0.0
Chongqing	4	17/17	0.37	6/6	11/11	2002-2009	15/15	2/2	94.1	0.0	0.0	0.0	5.9	0.0
Fujian	5	18/18	0.27	17/17	1/1	2002-2011	16/16	2/2	100.0	0.0	0.0	0.0	0.0	0.0
Gansu	1	3/3	0.00	0/0	3/3	2004-2004	3/3	0/0	100.0	0.0	0.0	0.0	0.0	0.0
Guangdong	53	211/186	14.64	164/145	47/41	2000-2015	46/35	165/151	94.3	0.5	0.0	0.0	4.7	0.5
Guangxi	17	60/57	17.27	37/34	23/23	2000-2014	24/23	36/34	88.3	0.0	0.0	3.3	8.3	0.0
Guizhou	2	4/4	0.04	1/1	3/3	2004-2013	3/3	1/1	100.0	0.0	0.0	0.0	0.0	0.0
Hainan	4	10/10	0.30	7/7	3/3	2002-2004	10/10	0/0	100.0	0.0	0.0	0.0	0.0	0.0
Hebei	1	3/3	0.02	0/0	3/3	2004-2004	3/3	0/0	100.0	0.0	0.0	0.0	0.0	0.0
Heilongjiang	10	34/32	36.24	11/11	23/21	2001-2012	30/29	4/3	67.7	0.0	2.9	29.4	0.0	0.0
Henan	3	21/21	0.10	3/3	18/18	2000-2011	20/20	1/1	100.0	0.0	0.0	0.0	0.0	0.0
Hubei	4	37/37	1.98	3/3	34/34	2000-2004	37/37	0/0	16.2	0.0	83.8	0.0	0.0	0.0
Hunan	8	65/60	22.78	52/47	13/13	2002-2012	25/25	40/35	100.0	0.0	0.0	0.0	0.0	0.0
Jiangsu	14	53/39	0.60	28/25	25/14	2000-2014	26/21	27/18	100.0	0.0	0.0	0.0	0.0	0.0
Jiangxi	3	9/9	0.08	6/6	3/3	2002-2004	9/9	0/0	100.0	0.0	0.0	0.0	0.0	0.0
Jilin	7	25/23	14.75	15/13	10/10	2002-2012	12/11	13/12	100.0	0.0	0.0	0.0	0.0	0.0
Liaoning	3	13/13	0.77	4/4	9/9	2004-2007	9/9	4/4	100.0	0.0	0.0	0.0	0.0	0.0
Inner Mongolia	0	0/0	-	0/0	0/0	-	0/0	0/0	-	-	-	-	-	-
Ningxia	0	0/0	-	0/0	0/0	-	0/0	0/0	-	-	-	-	-	-
Qinghai	0	0/0	-	0/0	0/0	-	0/0	0/0	-	-	-	-	-	-
Shaanxi	1	3/3	0.00	0/0	3/3	2004-2004	3/3	0/0	100.0	0.0	0.0	0.0	0.0	0.0
Shandong	10	36/34	0.06	13/13	23/21	2000-2012	22/20	14/14	88.9	0.0	2.8	0.0	8.3	0.0
Shanghai	1	3/3	0.00	0/0	3/3	2004-2004	3/3	0/0	100.0	0.0	0.0	0.0	0.0	0.0
Shanxi	1	3/3	0.00	0/0	3/3	2004-2004	3/3	0/0	100.0	0.0	0.0	0.0	0.0	0.0
Sichuan	4	24/22	0.11	2/2	22/20	2003-2012	18/16	6/6	100.0	0.0	0.0	0.0	0.0	0.0
Tianjin	2	8/5	0.18	0/0	8/5	2004-2004	8/5	0/0	100.0	0.0	0.0	0.0	0.0	0.0
Xinjiang Uygur	1	4/4	0.03	0/0	4/4	2004-2004	4/4	0/0	100.0	0.0	0.0	0.0	0.0	0.0
Tibet	0	0/0	-	0/0	0/0	-	0/0	0/0	-	-	-	-	-	-
Yunnan	2	9/9	0.00	6/6	3/3	2004-2004	9/9	0/0	100.0	0.0	0.0	0.0	0.0	0.0
Zhejiang	1	3/3	0.00	0/0	3/3	2004-2004	3/3	0/0	100.0	0.0	0.0	0.0	0.0	0.0
Total	143	691/633	9.69	376/344	315/289	2000-2015	375/363	316/288	90.5	0.1	4.8	1.7	2.8	0.1

^a^Presented as surveys/points.

^b^KK: Kato-Katz; Flot: stool floatation; Digest: sodium hydroxide (NaOH) digestion; Conc: stool concentration; Sedi: stool sedimentation; NS: not stated or missing.

### Variable selection, geostatistical modeling, and model validation

We considered a total of 12 variables (i.e., land cover, urban extents, precipitation, GDP per capita, HII, soil moisture, elevation, LST in the daytime, LST at night, NDVI, distance to the nearest open water bodies, and pH in water) for Bayesian variable selection. Elevation, NDVI, distance to the nearest open water bodies, and land cover were selected for the final geostatistical logistic regression model. The variables that were selected via the Bayesian variable selection method are listed in Supporting Information S1 Table. The list was not affected by the collinearity threshold (i.e., 0.5, 0.7, and 0.8) we have considered.

The parameter estimates arising from the geostatistical model fit are shown in Table 3. The infection risk of *C*. *sinensis* was higher from 2005 onwards than that before 2005. Elevation had a negative effect on infection risk. People living at distance between 2.5 and 7.0 km from the nearest open water bodies had a lower risk compared to those living in close proximity (<2.5 km). The risk of *C*. *sinensis* infection was lower in areas covered by forest, shrub, and grass compared to crop. Furthermore, NDVI was positively correlated with the risk of *C*. *sinensis* infection.

**Table 3:** Posterior summaries (median and 95% Bayesian credible intervals) of the geostatistical model parameters for clonorchiasis in P.R. China.

Variable	Estimate
Year	
2000–2004	0.00
≥2005	0.43 (0.38; 0.48)^a^
Elevation	-1.34 (-1.97; -0.83)^a^
NDVI	0.61 (0.41; 0.81)^a^
Distance to the nearest open water bodies (km)	
≤2.5	0.00
2.5–7.0	-0.46 (-0.72; -0.22)^a^
>7.0	0.05 (-0.38; 0.46)
Land cover	
Crop	0.00
Forest	-0.91 (-1.37; -0.42)^a^
Shrub and grass	-0.57 (-1.07; -0.16)^a^
Urban	0.06 (-0.36; 0.48)
Wet	0.24 (-0.39; 0.98)
Spatial range (km)	259.80 (199.70; 373.01)
$\sigma_{sp}^{2}$	13.64 (9.90; 20.51)

^a^Significant correlation based on 95% Bayesian credible interval.

Model validation indicated that the Bayesian geostatistical logistic regression models were able to correctly estimate (within a 95% BCI) 71.7% of locations for *C*. *sinensis*. The mean error was -0.07%, suggesting that our model may slightly over-estimate the infection risk of *C*. *sinensis*.

### Predictive risk maps and estimates of number of people infected

Fig 2A shows the model-based predicted risk map of *C*. *sinensis* for P.R. China. High prevalence (≥20%) was estimated in some areas of southern and northeastern parts of Guangdong province, southwestern and northern parts of Guangxi province, southwestern part of Hunan province, the western part of bordering region of Heilongjiang and Jilin provinces, and the eastern part of Heilongjiang province. Most regions of northwestern P.R. China and eastern coastal areas had zero to very low prevalence (<0.01%). The prediction uncertainty is shown in Fig 2C.

Table 4 reports the population-adjusted predicted prevalence and the number of individuals infected with *C*. *sinensis* in P.R. China, stratified by province, based on gridded population of 2010. The overall population-adjusted predicted prevalence of clonorchiasis was 1.18% (95% BCI: 1.10–1.25%) in 2010, corresponding to 14.8 million (95% BCI: 13.8–15.8 million) infected individuals. The three provinces with the highest infection risk were Heilongjiang (7.21%, 95% BCI: 5.95–8.84%), Guangdong (6.96%, 95% BCI: 6.62–7.27%), and Guangxi (5.52%, 95% BCI: 4.97–6.06%). Provinces with very low risk estimates (median predicted prevalence < 0.01%) were Gansu, Ningxia, Qinghai, Shanghai, Shanxi, Tibet, and Yunnan. Guangdong, Heilongjiang, and Guangxi were the top three provinces with the highest number of people infected: 6.34 million (95% BCI: 6.03–6.62 million), 3.05 million (2.52–3.74 million), and 2.08 million (1.87–2.28 million), respectively.

**Table 4:** Population-adjusted predicted prevalence and estimated number of people infected with *C*. *sinensis* in P.R. China, stratified by province in 2010^a^.

Province	Population (×10^6^)	Prevalence (%)^b^	No. infected (×10^3^)^b^
Anhui	54.89	0.66 (0.51; 0.89)	363.67 (279.09; 489.63)
Beijing	16.99	0.02 (0.00; 0.11)	2.64 (0.20; 17.98)
Chongqing	26.72	0.13 (0.09; 0.20)	35.96 (23.52; 52.15)
Fujian	32.80	0.09 (0.05; 0.17)	29.33 (17.08; 56.51)
Gansu	25.60	0.00 (0.00; 0.00)	0.10 (0.01; 0.88)
Guangdong	91.06	6.96 (6.62; 7.27)	6341.38 (6030.46; 6622.18)
Guangxi	37.62	5.52 (4.97; 6.06)	2077.94 (1867.98; 2280.70)
Guizhou	31.37	0.05 (0.02; 0.08)	15.33 (7.58; 26.67)
Hainan	6.68	0.46 (0.26; 0.71)	30.42 (17.05; 47.62)
Hebei	75.52	0.04 (0.02; 0.09)	32.20 (13.77; 67.43)
Heilongjiang	42.28	7.21 (5.95; 8.84)	3050.57 (2515.22; 3737.51)
Henan	84.30	0.11 (0.07; 0.16)	91.32 (60.46; 138.85)
Hubei	58.21	2.26 (1.90; 2.66)	1313.52 (1103.91; 1548.28)
Hunan	55.14	0.61 (0.51; 0.75)	333.64 (283.77; 410.98)
Jiangsu	74.30	0.19 (0.15; 0.24)	144.31 (113.62; 177.60)
Jiangxi	36.26	0.15 (0.09; 0.25)	54.72 (31.76; 91.40)
Jilin	29.19	2.07 (1.76; 2.43)	605.43 (514.64; 707.94)
Liaoning	43.09	0.32 (0.24; 0.45)	139.05 (104.56; 194.56)
Inner Mongolia	29.73	0.08 (0.05; 0.12)	24.09 (14.87; 37.06)
Ningxia	6.27	0.00 (0.00; 0.00)	0.03 (0.01; 0.18)
Qinghai	4.96	0.00 (0.00; 0.00)	0.00 (0.00; 0.01)
Shaanxi	34.26	0.01 (0.00; 0.03)	2.32 (0.26; 11.53)
Shandong	93.37	0.04 (0.03; 0.07)	41.06 (24.86; 65.24)
Shanghai	14.95	0.00 (0.00; 0.04)	0.36 (0.01; 5.34)
Shanxi	35.45	0.00 (0.00; 0.02)	1.38 (0.22; 5.39)
Sichuan	94.63	0.04 (0.02; 0.06)	34.48 (20.55; 57.33)
Tianjin	9.76	0.03 (0.02; 0.06)	2.97 (1.58; 5.41)
Xinjiang Uygur	24.97	0.01 (0.00; 0.06)	3.58 (1.10; 14.62)
Tibet	2.68	0.00 (0.00; 0.00)	0.00 (0.00; 0.00)
Yunnan	39.49	0.00 (0.00; 0.01)	1.31 (0.30; 4.24)
Zhejiang	45.35	0.02 (0.00; 0.08)	8.31 (1.04; 36.06)
Total	1257.89	1.18 (1.10; 1.25)	14844.08 (13796.77; 15767.23)

^a^Estimates based on gridded population of 2010; calculations based on the median and 95% Bayesian credible interval of the posterior distribution of the predictive risk from 2005 onwards.

^b^Summarized as median and 95% Bayesian credible interval.

## Discussion

To our knowledge, we present the first model-based, high-resolution estimates of *C*. *sinensis* infection risk in P.R. China. Risk maps were produced through Bayesian geostatistical modeling of clonorchiasis survey data from 2000 onwards, readily adjusting for environmental/climatic predictors. Our methodology is based on a rigorous approach for spatially explicit estimation of neglected tropical disease risk [27]. Surveys pertaining to prevalence of *C*. *sinensis* in P.R. China were obtained through a systematic review in both Chinese and worldwide scientific databases to obtain published work from 2000 onwards. Additional data were provided by the NIPD, China CDC.

We estimated that 14.8 million (95% BCI: 13.8–15.8 million; 1.18%) people in P.R. China were infected with *C*. *sinensis* in 2010, which is almost 20% higher than the previous estimates of 12.5 million people for the year 2004, based on empirical analysis of data from a large survey of clonorchiasis conducted from 2002–2004 in 27 endemic P/A/M. The mean error for the model validation was slightly smaller than zero, suggesting that our model might somewhat over-estimate the true prevalence of clonorchiasis. The overall raw prevalence of the observed data was 9.7%. This can be an over-estimation of the overall prevalence as many surveys were likely to have been conducted in places with relatively high infection risk (preferential sampling). Our population-adjusted, model-based estimates was much lower (1.18%, 95% BCI: 1.10–1.25%) and it should reflect the actual situation because it takes into account the distribution of the population and of the disease risk across the country. Indeed, geostatistical models get their predictive strength from regions with large amount of data that allow more accurate estimation of the relation between the disease risk and its predictors, therefore they are the most powerful statistical tools for predicting the disease risk in areas with sparse data. Still, the estimates in regions with scarce data should be interpreted with caution. However, even though our data did not include surveys from four provinces (Inner Mongolia, Ningxia, Qinghai, and Tibet), our model obtained low or zero prevalence estimates which are consistent with data summaries of the second national survey aggregated at provincial level for these four provinces [7]. On the other hand, our model may overestimate the overall infection risk for Heilongjiang province, as the high risk areas in the southeastern and southwestern parts of the province may influence the prediction in the northern part, where no observed data were available.

We found an increase of infection risk of *C*. *sinensis* for the period from 2005 onwards, which may be due to several reasons, including higher consumption of raw fish, lack of self-protection awareness of food hygiene, low health education, and rapid growth of aquaculture [13,36]. Consumption of raw freshwater fish is related to *C*. *sinensis* infection risk [15,37], however, such information is unavailable for P.R. China.

Elevation was one of the most important predictors in our model. Different elevation levels correspond to different environmental/climatic conditions that can influence the distribution of intermediate host snails. Our results show a positive association of NDVI and the prevalence of *C*. *sinensis*. We found that distance to the nearest water bodies was significantly related to infection risk. Traditionally, areas adjacent to water bodies were reported to have a higher prevalence of *C*. *sinensis*, however, due to improvement of trade and transportation channels, this situation may be changing, which may explain our result showing a non-linear relationship between distance to nearest water bodies and infection risk [2,13]. Furthermore, our analysis supports earlier observations, suggesting an association between land cover type and infection risk [13,14].

Interestingly, the risk of infection with other neglected tropical diseases, such as soil-transmitted helminthiasis and schistosomiasis, has declined in P.R. China over the past 10–15 years due to socioeconomic development and large-scale interventions [38]. However, clonorchiasis, the major food-borne trematodiases in P.R. China, shows an increased risk in recent years, which indicates the Chinese government needs to pay more attention to this disease. Several areas with high infection risk in P. R. China are indicated (Supporting Information S2 Fig), where control strategies should be focused.

The recommended treatment guidelines for clonorchiasis of the WHO advocate praziquantel administration for all residents every year in high endemic areas (prevalence ≥20%) and for all residents every two years or individuals regularly eating raw fish every year in moderate endemic areas (prevalence <20%) [19]. As re-infection or super-infection is common in heavy endemic areas, repeated preventive chemotherapy is necessary to interrupt transmission [18]. On the other hand, to maintain control sustainability, a comprehensive control strategy must be implemented, including IEC, preventive chemotherapy, and improvement of sanitation [20,21]. Through IEC, residents may conscientiously reduce or stop consumption of raw fish. Furthermore, by removing unimproved latrines around fish ponds, the likelihood of fish becoming infected with cercariae declines [39]. A successful example of comprehensive control strategies is Shangdong province, where clonorchiasis was endemic, but after rigorous implementation of comprehensive control programs for more than 10 years, the disease has been well controlled [40].

The Chinese Ministry of Health set a goal to halve the prevalence of clonorchiasis (compared to that observed in the second national survey in 2001–2004) in highly endemic areas by 2015 using integrated control measures [30]. In practice, control measures are carried out in endemic villages or counties with available survey data. However, large-scale control activities are lacking in most endemic provinces, as control plans are difficult to make when the epidemiology is only known at provincial level [41]. Our high-resolution infection risk estimates provide important information for targeted control.

Our analysis is based on historical survey data compiled from studies that may differ in study design, diagnostic methods and distribution of age groups. As more than 90% of surveys applied Kato-Katz as diagnostic method, we assumed similar diagnostic sensitivity across all surveys. However, the sensitivity may vary in space as a function of infection intensity. Most of the survey data are aggregated over age groups, thus we could not obtain age-specific risk estimates. Moreover, bias might occur when age distribution in survey population differ across locations as different age group may have different infection risk.

In conclusion, we present the first model-based, high-resolution risk estimates of *C*. *sinensis* infection in P.R. China, and identified areas of high priority for control. Our findings show an increased risk from 2005 onwards, suggesting that the government should put more efforts on control activities of clonorchiasis in P.R. China.

## Supporting information

S1 Checklist PRISMA Checklist. (DOC)

S1 Text Simulation Study. (DOCX)

S1 Table Posterior Inclusion Probabilities for the Variables Assessed in the Bayesian Variable Selection Procedure. (DOCX)

S1 Fig Predictive Ability of Models on Simulation Study. (A) and (C) show the mean absolute error and log score for each dataset, respectively. (B) and (D) depict the distribution of the mean absolute error and log score overall all datasets by each model, respectively.
(DOCX)

S2 Fig High Infection Risk Areas (Median of Posterior Predictive Distribution of Prevalence >20%) in the Corresponding Provinces. (DOCX)
