# Supplementary material for: Risk mapping of clonorchiasis in the People’s Republic of China: A systematic review and Bayesian geostatistical analysis
Source: PLoS Negl Trop Dis. 2017 Mar 2;11(3):e0005239. doi: 10.1371/journal.pntd.0005239 (PMC5416880; doi:10.1371/journal.pntd.0005239)
Supplement: S1 Text — (DOCX) [file pntd.0005239.s002.docx]

S1 Text. Simulation Study.

We carried out a simulation study to assess the predictive ability of geostatistical models that combine both point-referenced and areal survey data, by setting the locations of the areas (e.g., counties) at their centroids (i.e., model 1) compared to models that include only point-referenced data (i.e., model 2) for analysis.

We avoided high computation burden, by considering in the simulation only two neighboring provinces, Guangdong and Guangxi. 30 datasets were generated following the structure of the survey data in the region. We randomly sampled 12 points within each survey county which we treated them as the “unknown” survey locations in the county. A Gaussian process was generated over the whole set of locations (both observed and “unknown”), that is $\boldsymbol{w}={(w_{1},\ldots,w_{s})}^{T}$, $\boldsymbol{w}\sim MVN(0,\Sigma)$, and $\Sigma_{ij}=\sigma^{2}exp(-\rho d_{ij})$, where $d_{ij}$ is the Euclidean distance between locations $i$ and $j$. We set $\sigma^{2}=2$ and $\rho=5$. We considered a single covariate $\boldsymbol{X}$ corresponding to the elevation and simulated survey data (i.e., number of infected individuals at location $i$) from a binomial distribution $y_{i}\sim Bin\left( p_{i},n_{i} \right)$ with binomial proportion $p_{i}=\mathrm{logit}^{-1}(\beta_{0}+\beta_{1}X_{i}+w_{i})$ by setting $\beta_{0}=-2$ and $\beta_{1}=-2$ and by assuming equal number of screened individuals $n_{i}=500$ across locations. The simulated areal data was calculated as $Y_{k}=\sum_{i\in A_{k}} y_{i}$ and $N_{k}=\sum_{i\in A_{k}} n_{i}$, where $Y_{k}$, $N_{k}$ and $A_{k}$ represent the number of infected, number of screened individuals and all the sampled points in county $k$. Simulations were carried out in R version 3.3.2.

The model fitting procedure is the same to the one presented in the statistical analysis section in the main text. We randomly selected 20% of point-referenced and of sampled within areas points for model validation. The remaining ones were for model fit. The mean absolute error (MAE) and predictive logarthmic score [1] were used for model validation. Lower MAE and higher logarithmic score suggest a model with better predictive ability.

The results from the simulation study are shown in Supporting Information S2 Fig. For better visualization, we connected datasets of the same model with lines in S2 Fig A and C. Model 1 had lower MAE and higher log score than that of model 2, suggesting a better predictive ability.

Reference

1. Gneiting T, Raftery AE. Strictly proper scoring rules, prediction, and estimation. J Am Stat Assoc. 2007; 102: 359-378.
