# Supplementary material for: Risk mapping of clonorchiasis in the People’s Republic of China: A systematic review and Bayesian geostatistical analysis
Source: PLoS Negl Trop Dis. 2017 Mar 2;11(3):e0005239. doi: 10.1371/journal.pntd.0005239 (PMC5416880; doi:10.1371/journal.pntd.0005239)
Supplement: S1 Table — (DOCX) [file pntd.0005239.s003.docx]

S1 Table. Posterior Inclusion Probabilities for the Variables Assessed in the Bayesian Variable Selection Procedure.

| Variables | | | Posterior mean of indicators | Selected^*^ |
| --- | --- | --- | --- | --- |
| Land cover | Croplands | | 1.00 | Yes |
|  | Forest | |  |  |
|  | Shrublands and grass | |  |  |
|  | Urban | |  |  |
|  | Wet areas | |  |  |
| Urban extends | rural | | 0.31 | No |
|  | urban | |  |  |
| Annual precipitation | Continuous | | 0.02 | No |
|  | Categorical | ≤1,200 mm | 0.03 | No |
|  |  | >1,200 mm |  |  |
| GDP per capita | Continuous | | 0.10 | No |
|  | Categorical | ≤20,000 yuan | 0.02 | No |
|  |  | >20,000 yuan |  |  |
| HII | Continuous | | 0.01 | No |
|  | Categorical | ≤30 | 0.13 | No |
|  |  | >30 |  |  |
| Soil moisture | Continuous | | 0.29 | No |
|  | Categorical | ≤100 mm | 0.18 | No |
|  |  | >100 mm |  |  |
| Elevation | Continuous | | 1.00 | Yes |
|  | Categorical | ≤100 m | 0.00 | No |
|  |  | 100-500 m |  |  |
|  |  | >500 m |  |  |
| NDVI | Continuous | | 1.00 | Yes |
|  | Categorical | ≤0.45 | 0.00 | No |
|  |  | 0.45-0.55 |  |  |
|  |  | >0.55 |  |  |
| Distance to the nearest waterbodies | Continuous | | 0.00 | No |
|  | Categorical | ≤2.5 km | 0.99 | Yes |
|  |  | 2.5-7.0 km |  |  |
|  |  | >7.0 km |  |  |
| PH in water | Continuous | | 0.23 | No |
|  | Categorical | ≤6 | 0.00 | No |
|  |  | 6-7 |  |  |
|  |  | >7 |  |  |
| LST at day | Continuous | | 0.03 | No |
|  | Categorical | ≤18 ℃ | 0.07 | No |
|  |  | 18-23 ℃ |  |  |
|  |  | >23 ℃ |  |  |
| LST at night^#^ | | | - | - |

^*^Variables with inclusion probabilities higher than 50% were included in the final geostatistical model; ^#^dropped due to high correlation with LST at day and annual precipitation (i.e., correlation coefficient >0.8).
