# Supplementary material for: Risk mapping of clonorchiasis in the People’s Republic of China: A systematic review and Bayesian geostatistical analysis
Source: PLoS Negl Trop Dis. 2017 Mar 2;11(3):e0005239. doi: 10.1371/journal.pntd.0005239 (PMC5416880; doi:10.1371/journal.pntd.0005239)
Supplement: S1 Fig — (A) and (C) show the mean absolute error and log score for each dataset, respectively. (B) and (D) depict the distribution of the mean absolute error and log score overall all datasets by each model, respectively. (DOCX) [file pntd.0005239.s004.docx]

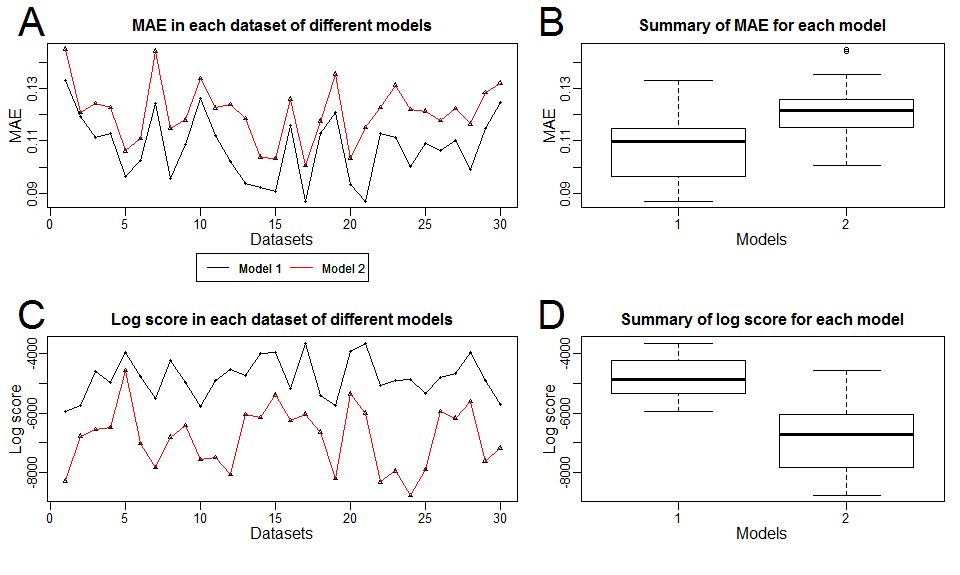


S1 Fig. Predictive Ability of Models on Simulation Study. (A) and (C) show the mean absolute error and log score for each dataset, respectively. (B) and (D) depict the distribution of the mean absolute error and log score overall all datasets by each model, respectively.
