# Supplementary material for: Risk mapping of clonorchiasis in the People’s Republic of China: A systematic review and Bayesian geostatistical analysis
Source: PLoS Negl Trop Dis. 2017 Mar 2;11(3):e0005239. doi: 10.1371/journal.pntd.0005239 (PMC5416880; doi:10.1371/journal.pntd.0005239)
Supplement: S2 Fig — (DOCX) [file pntd.0005239.s005.docx]

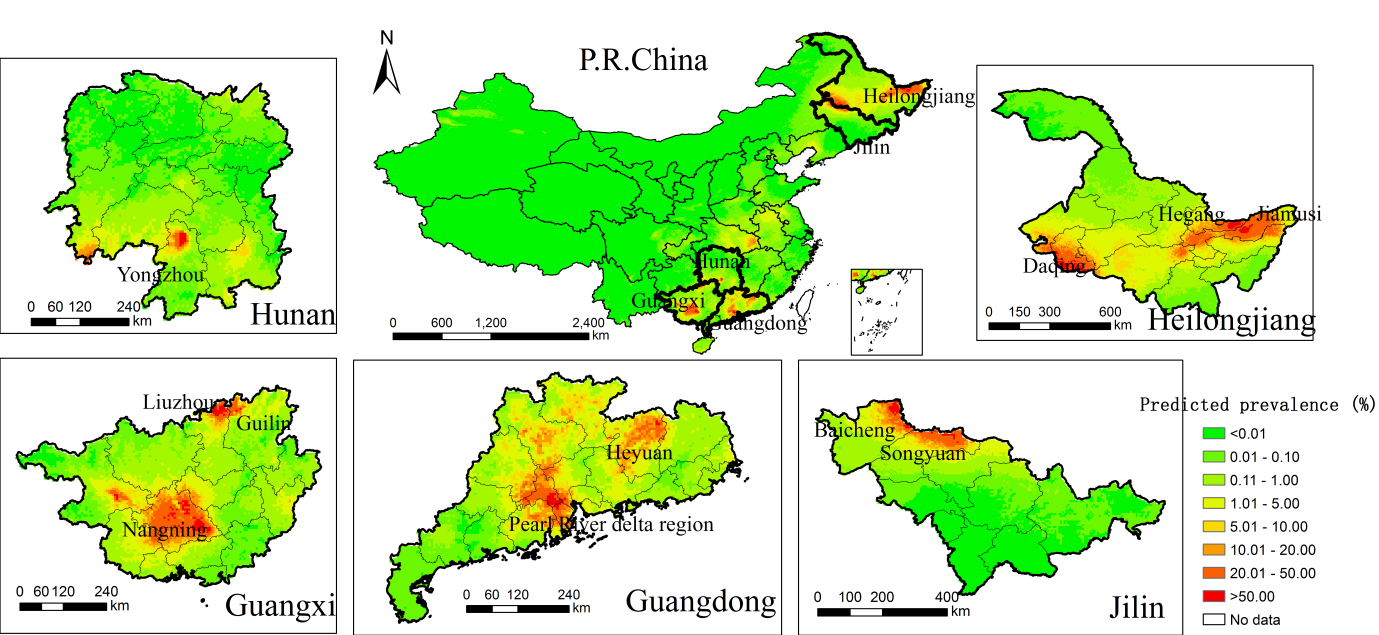


S2 Fig. High Infection Risk Areas (Median of Posterior Predictive Distribution of Prevalence >20%) in the Corresponding Provinces.
